# Websites about, not for, adolescents? A systematic analysis of online fertility preservation information for adolescent and young adult cancer patients

**DOI:** 10.21203/rs.3.rs-2587513/v1

**Published:** 2023-02-16

**Authors:** Sienna Ruiz, Rachel Mintz, Amela Sijecic, Michelle Eggers, Aubri Hoffman, Terri Woodard, Kari Louise Bjonard, Holly Hoefgen, Taryn Sandheinrich, Kenan Omurtag, Ashley J. Housten

**Affiliations:** Washington University in St. Louis; Washington University in St. Louis; Washington University in St. Louis; Washington University in St. Louis; The University of Texas at Austin; The University of Texas MD Anderson Cancer Center; Indiana University; Washington University in St. Louis; Washington University in St. Louis; Washington University in St. Louis; Washington University in St. Louis

**Keywords:** adolescent, cancer, comprehension, decision making – shared, fertility, fertility preservation, internet, patient education, survivorship, young adult

## Abstract

**Purpose:**

Fertility preservation is an increasingly important topic in adolescent and young adult cancer survivorship, yet treatments remain under-utilized, possibly due to lack of awareness and understanding. The internet is widely used by adolescents and young adults and has been proposed to fill knowledge gaps and advance high-quality, more equitable care. As a first step, this study analyzed the quality of current fertility preservation resources online and identified opportunities for improvement.

**Methods:**

We conducted a systematic analysis of 500 websites to assess the quality, readability, and desirability of website features, and the inclusion of clinically relevant topics.

**Results:**

The majority of the 68 eligible websites were low quality, written at college reading levels, and included few features that younger patients find desirable. Websites mentioned more common fertility preservation treatments than promising experimental treatments, and could be improved with cost information, socioemotional impacts, and other equity-related fertility topics.

**Conclusions:**

Currently, the majority of fertility preservation websites are about, but not for, adolescent and young adult patients. High-quality educational websites are needed that address outcomes that matter to teens and young adults, with a priority on solutions that prioritize equity.

**Implications for Cancer Survivors:**

Adolescent and young adult survivors have limited access to high-quality fertility preservation websites that are designed for their needs. There is a need for the development of fertility preservation websites that are clinically comprehensive, written at appropriate reading levels, inclusive, and desirable. We include specific recommendations that future researchers can use to develop websites that could better address AYA populations and improve the fertility preservation decision making process.

## Introduction

Every year, more than 85,000 adolescents or young adults (AYAs; or patients between the ages of 12–39) are diagnosed with cancer in the United States [1,2]. While survival rates for AYAs with cancer are high [2], many survivors experience infertility from the toxicities of their cancer and treatments [3,4]. The American Society for Clinical Oncology (ASCO) recommends that providers discuss the options for fertility preservation with all patients of reproductive age [5]. However, fertility preservation services remain under-prescribed and under-utilized [6], and survivors report high levels of infertility-related distress and regret [7]. AYAs report low awareness and knowledge as a critical barrier for engaging in fertility preservation decision making [8].

Although AYA’s participation in treatment decisions is essential [9], providers and parents may face structural and social challenges to incorporating AYA participation in their cancer treatment decision making, which can limit opportunities to help them become aware and well-informed about their treatment options [10]. This may be due to short windows of time for clinical visits or perceptions about adolescents’ maturity levels or rationality that can result in education and communication focused on parents and caregivers rather than AYA patients [9–12]. Engaging AYAs in treatment decision making can also be difficult as AYAs are simultaneously navigating developmental milestones, school or early employment [13], dynamic social lives [14], potentially variable insurance coverage [15], and embarrassment surrounding topics related to fertility [16]. These challenges can augment existing difficulties surrounding discussions on fertility preservation for AYAs with cancer. Younger AYA patients report challenges in finding age-appropriate online resources about cancer-related topics [27], and specific to fertility preservation, they describe limited opportunities for clinical conversations on treatment options [7,17], incomplete understandings of their fertility risk [18], large psychological impacts of fertility preservation [19], and worries about the financial resources needed for to treatment [20]. Due to these limitations and clinical eligibility for fertility preservation treatments for children both pre- and post-puberty [21], for this study, we were particularly interested in resources for resources for younger AYAs and therefore focused our research question on resources for those in the younger AYA age range..

AYA knowledge and empowerment related to fertility preservation decision making is critical and could potentially be aided through the use of relevant, accessible, accurate and trustworthy online resources. Providing high-quality patient education resources online can improve access to trusted information between visits. Online resources are widely used among AYA patients [22,23], with more than 95% of young adults having access to online information [24]. Furthermore, younger patients and many marginalized groups use health care websites as a primary source of health information [25] but report difficulties navigating large amounts of online resources [26], providing an opportunity to address equity gaps in fertility care [8,27].

Building on past research examining internet-based fertility preservation information for adult cancer patients [28,29], this review seeks to characterize the quality, desirability, and content of existing online fertility preservation information for AYA cancer patients. It also contextualizes current online information and identifies opportunities for improvement.

## Methods

### Website Selection

In collaboration with clinicians, decision scientists, health literacy experts, and a clinical librarian, we followed the Preferred Reporting Items for Systematic Reviews and Meta-Analyses (PRISMA) guidelines. To mimic AYA patients’ and caregivers’ internet search behavior, we used multiple patient-friendly search phrases in the two most-used search engines: Google and Yahoo [30–32]. We developed five search phrases: “fertility preservation cancer teen;” “fertility preservation cancer adolescent;” “fertility preservation cancer young adult;” “egg freezing cancer teen;” and “sperm banking cancer teen”. Our discussions with a clinical librarian informed our use of the phrase “teen” in addition to “adolescent” and “young adult” rather than more medically oriented terms like “AYA” to represent common language patients and their families might use to represent younger AYA patients. We also specified the terms “egg freezing” and “sperm banking” as they are among the most common forms of fertility preservation known by patients and families as well as the most widely available and efficacious treatments that patients would likely be offered by providers.

Before searching each term, reviewers cleared their cache, cookies, and search and usage history to depersonalize the search and reduce bias. We reviewed the first 50 results generated on each search engine for each phrase. After obtaining 50 results for each search phrase, we cross-referenced the results to remove any duplicate websites. We then excluded websites that were not intended to provide patient education for the general public, such as academic publications, resources for medical professionals, treatment guidelines, and information on clinical trials. In addition, we excluded advertisements, websites not written in English, news articles from popular media sources (e.g. from non-medically oriented popular media like lifestyle magazines or news outlets) or about academic articles or treatment guidelines, blogs with personal narratives written in the first or third person with fertility preservation, social media posts, podcasts, presentations, PDFs or documents, and content “tags” used to filter posts on larger websites. We excluded PDFs, documents, and presentation because we felt that they could not be evaluated against interactive websites and would be unfairly penalized for lacking features like that are not possible to include in these forms of online resources.

We also excluded websites that did not include key terms relevant to AYAs and fertility preservation. Sites were considered relevant to AYAs if they referred to patients between the ages of 12–25 years old or contained the words “child/children”, ““adolescent,” “young adult” or its abbreviation YA, “teen,” “pediatric,” “girl” or “boy” in the site title or the text of the website. We only considered the terms “child/children,” “girl,” and “boy” relevant if they referred to the patient potentially undergoing fertility preservation, not the patient’s ability to have a child. We also considered sites relevant if they included discussions of puberty or menarche, or words related to fertility or fertility preservation (e.g., “egg freezing”, “oocyte cryopreservation,” “sperm banking,” “sperm cryopreservation,” “ovarian tissue cryopreservation,” “menstrual suppression,”).

## Website Characteristics And Evaluation

All eligible websites were recorded in an internal data extraction sheet using REDCap [33,34] hosted at Washington University School of Medicine. Core members of the research team (SR, RM, AS, AJH) worked together to identify the tools to summarize the content of these websites and to create an initial coding manual. The full team reviewed the approach and provided feedback on the codebook. The core team coded the first three records together and continued until all coders reached consensus. A minimum of two independent coders (SR, RM, AS) extracted the website links, titles, and ranked positions in search results (i.e., the number out of 50 in which the website appeared). Coders also qualitatively tracked the website’s intended audience as For Parents or Caregivers, For AYA Patients, or For Both Audiences based on the language used by each site. For example, if the site referred to “your child” or directly referenced the thoughts, concerns, or questions of parents or caregivers, we coded it as aimed at the parents or caregivers of AYA patients diagnosed with cancer.

To evaluate each website, we used a combination of validated measures and team-created evaluation tools to characterize the website’s quality, readability, desirability, and inclusion of topics recommended by National Comprehensive Cancer Network (NCCN) practice guidelines for AYA oncology patients.

To measure the quality of websites on fertility preservation for AYA patients, we used DISCERN, a validated measure of quality for online health information [30]. DISCERN records whether written health materials cite outside sources of information, provide an impartial assessment of all treatment choices, and support shared decision making, among other elements. Two independent coders answered each of the 16 questions as 1 No, 2 to 4 Partially, or 5 Yes. To distinguish between partial scores, websites received a 2, 3, or 4 if they complete one, two, or three of the hints provided by the DISCERN online rating guide [35], respectively. Scores were summed to create a total score for each website. Two independent coders reconciled their scores, discussed any discrepancies to reach consensus, and rated sites using DISCERN categories of excellent (scores of 63–75), good (51–62), fair (39–50), poor (27–38), and very poor (16–26; 24,25) quality.

Next, we used the Simple Measure of Gobbledygook (SMOG; 27) readability formula due to its ability to measure reading levels below the 12th grade reading level. To minimize human error, we used the automatic Uniform Resource Locator (hereafter referred to as URL) scoring feature. This online readability calculator automatically excludes headers, coding, and sidebar text and extracts only the relevant text from the websites to determine readability scores. Coders reviewed the automatically generated scores by examining the online calculator’s extracted text of every website for any extraneous errors (e.g., coding, headers, URLs to other websites, and alternate spacing that can artificially alter the SMOG score). If the text that the calculator scored contained errors, a coder copied and pasted text from a “reader view” of the web page (which only shows the main text with no headers or sidebars) into the manual text scoring function of the online readability calculator. Then the team compared the manual score of the text to the original online calculator score with errors, and recorded the manual score if there was a difference greater or equal to 0.5 (i.e., half of a grade year). For ease of analysis, we labeled scores above the 12th grade as College Level.

To assess the desirability of the websites, we used Schiffman et al.’s desirability measure [23]. Schiffman et al. elicited AYA patients’ preferences regarding what they would like to see in patient-facing cancer education websites, and used their responses to create a list of 21 questions on the inclusion of features patients identified as desirable [23]. We modified the measure slightly to refer specifically to fertility preservation websites, and used it to record how many features each website included as the website’s desirability score.

To record websites’ inclusion of topics recommended by practice guidelines for patient-provider discussions on fertility preservation, we created a checklist of topics based on the NCCN Clinical Practice Guidelines in Oncology Adolescent and Young Adult (AYA) Oncology Version 2.2022 [39]. Each suggested topic to be covered in a clinical visit was converted into a Yes/No question representing presence or absence of a topic on the website. Although websites and online information are not replacements for clinical visits and have different informational parameters than clinical encounters, we aimed to record whether the topics addressed by websites aligned with current standards for medical practice. The number of topics on each website was added for a total score for our checklist of NCCN-recommended topics.

For a table of all of the measures we used for this research, please see Appendix 1.

## Results

**Figure 1:**
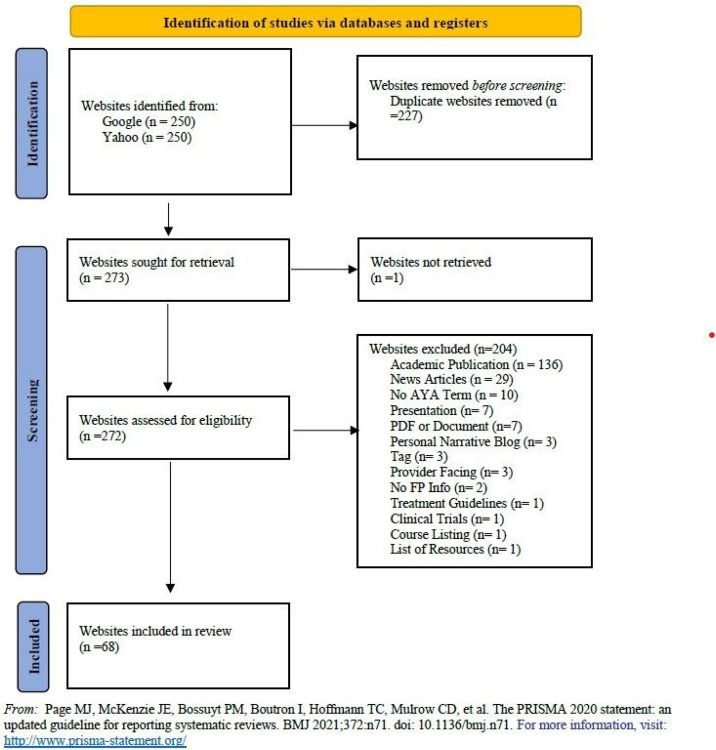
PRISMA Diagram

Our search strategies yielded 500 sites for evaluation. After excluding 227 duplicate websites, we assessed 273 websites for eligibility. Following eligibility screening, we determined 68 websites to be eligible for review (PRISMA diagram, Fig. 1). For a full breakdown of the review results, please see the Appendices 2–4.

Most of the 68 eligible websites were from general or pediatric hospitals (non-NCI Cancer Centers; n = 24, 35.3%) or cancer-related non-profits (22%, n = 15). Other websites were NCI Designated Comprehensive Cancer Centers (n = 9, 13.2%), fertility centers and sperm banks (n = 7, 10.3%), independent (e.g. non-academic, non-practice oriented) patient-facing websites or publications (n = 6, 8.8%), academic institutions (n = 5, 7.4%), and practitioner or research-based societies and institutions (i.e., ASCO or NIH; n = 2, 2.9%).

On average, these websites appeared as the 28th search result. Thirty-one websites (45.6%) were intended for AYA patients, 28 (41.2%) were intended for both patients and parents, and nine websites (13.2%) were solely aimed at parents or caregivers of AYA patients. Less than half (n = 31; 46%) of websites were solely dedicated to AYA patients.

## Quality

According to the DISCERN measure of quality, the websites scored as Very Poor (n = 15, 22.1%), Poor (n = 29, 42.6%), Fair (n = 17, 25%), Good (n = 6, 8.8%), Excellent (n = 1, 1.5%) quality.

In terms of DISCERN questions on sites’ content, 57 websites (83.8%) scored low (i.e., 1–3, Appendix 2) to the question, “Does it refer to areas of uncertainty?”, because they contained little information on uncertainty related to the experimental nature of certain treatment choices, unclear success rates for fertility preservation procedures in terms of successful collection of biological samples or a successful future attempt at pregnancy, or unknown benefits and risks of various procedures. For the question, “Is it clear what sources of information were used to compile the publication (other than the author or producer)?, the majority of sites scored low (n = 66, 97%), meaning they had few or no references to sources other than the author or producer. In response to the question, “Is it balanced and unbiased?”, nearly three quarters of sites (n = 47, 69.1%) scored low because they did not clearly indicate whether they were written from a personal or objective point of view, there was no evidence that a range of sources were used to inform the content, and there was no evidence of external review.

## Readability

All websites were at or above a 9th grade reading level, and the average reading level for all websites was 12th grade. About half (n = 32, 47.1%) were at a college reading level (grades 13–14). Less than 10% (n = 5, 7.4%) were at a 9th grade reading level, 10.3% (n = 7) were at a 10th grade reading level, 10.3% (n = 7) at an 11th grade reading level, and 25% (n = 17) at a 12th grade reading level.

## Desirability

The websites included an average of two out of the 21 AYA-generated desirable features. About a third of websites had one feature (n = 21, 30.9%) and (n = 2, 2.9%) had no features. The most common feature included was facts about fertility preservation, with almost all websites containing that information (n = 63, 92.6%). Almost half (n = 29, 42.6%) of websites included links to other fertility preservation sites or resources. Less than 20% of websites (n = 12, 17.6%) included survivor stories, had a question and answer section (16.2%, n = 11), or contained animations or videos (n = 12, 17.6%).

## Inclusion Of Nccn Practice Guideline Topics

Of the 68 eligible sites, 13.2% (n = 9) were written solely for male patients, 29.4% (n = 20) were written for female patients, and 57.4% (n = 39) included both male and female content. Of the 48 websites with content for male patients, 89.5% (n = 43) mentioned sperm banking, 16.7% (n = 8) mentioned cyclic guanosine monophosphate-specific phosphodiesterase type 5 (PDE5) inhibitors or electro-ejaculation for patients with erection or ejaculation issues, and 14.6% (n = 7) mentioned testicular sperm extraction (TESE). Of the 59 sites with content for female patients, 88.1% (n = 52) mentioned oocyte or embryo cryopreservation, 59.3% (n = 35) mentioned ovarian tissue cryopreservation, 28.8% (n = 17) mentioned menstrual suppression through medications like GnRH agonists, and 39% (n = 23) mentioned oophorexy or ovarian transposition.

Related to socioemotional topics recommended by NCCN, 39.7% (n = 27) of the 68 websites discussed costs related to fertility preservation, and few (n = 18, 26.5%) mentioned psychological or emotional considerations of fertility preservation. Notably, only 5.9% (n = 4) addressed additional complications for fertility preservation for transgender patients.

## Discussion

Overall, websites dedicated to fertility preservation for AYA patients were of lower quality and written at a high reading level, with few features that AYA patients find desirable and few topics recommended by NCCN practice guidelines. To address gaps in knowledge and foster engagement in shared decision making, high quality, accessible, and inclusive fertility preservation resources are needed that are attractive and engaging for AYA populations.

Only 46% of websites were solely dedicated to AYA patients. While discussion of fertility preservation undoubtedly involves both AYA patients and caregivers, there is also a need for patient-centered communication designed specifically for the needs of AYA patients, especially given that they are the patients that may undergo the treatments and will live most directly with the impacts of such treatments. Since our analysis revealed that many websites were not solely for AYA patients, and that websites were generally written at a high reading level with few features AYAs might find desirable, it appears that the sites in this search were functionally about, not designed for AYA patients. While most websites had facts about fertility preservation, few included desirable elements for adolescents and young adults, which may make fertility preservation information appear uninteresting or irrelevant. Furthermore, most sites would appear on the second or third page of search results and may be overlooked by AYA patients who typically consider the first or second search results the most trustworthy [40].

Low DISCERN scores indicate that much of the online information for AYA patients may have significant quality limitations that could exacerbate knowledge gaps [8]. Many websites scored poorly on conveying uncertainty, meaning that AYA patients are likely not to learn about the uncertainty of the effectiveness of future fertility procedures (e.g., in-vitro fertilization), the experimental nature of certain treatments, or unknown upfront benefits and risks of specific treatments. Low DISCERN scores on questions of bias also showed that many websites were potentially biased either towards specific treatments or specific institutions, and scores on sourcing showed that sites had few external sources for their informational content. Without external sources, AYAs may be deterred from reviewing source documentation to support the credibility of information presented on the website, which is a central tenant of online literacy.

The high reading levels (average 12th grade) of these websites underscore the inaccessibility of online content on fertility preservation, for adults and for AYAs. The average reading level for adults is 8th grade [41,42], and adolescence begins in the 6th grade. Appropriate and clearly written health information has the potential to increase patients’ knowledge of their own health conditions and treatments, prepare patients to better advocate for themselves, and improve the quality of patients’ medical decisions [43–45]. Previous studies have shown that features such as survivors’ narratives or videos can help patients with low health literacy better understand health information [46,47].For fertility preservation, which is a complex topic involving multiple treatment options related to sexual health and reproductive functions that can be difficult to understand, supporting patient knowledge by using such features

The results of this study also align with the extant literature showing that the majority of fertility preservation websites largely focus on common fertility preservation treatments like egg or embryo freezing and sperm banking and mostly focus only on the procedure. This gap observed in the results of our review suggests a lack information on promising emerging treatments, socioeconomic issues, psychological or emotional experiences, or gender identity. Notably, few websites discussed PDE5 inhibitors or electro-ejaculation, potentially overlooking patients that are peri-pubertal or that have physical or psychological difficulties with sample production [48]. In the context of socioeconomic issues, less than half (39.7%) of the websites included costs, a significant barrier to fertility preservation [49] and an increasingly important topic to patients and providers [50–52]. Financial hardship related to the cost of fertility preservation can dissuade patients from treatments [8] or cause financial difficulties later on in patients’ lives [53], meaning that common online sources may exclude discussion of a significant topic that can have wide-reaching impacts for long-term quality of life among AYAs. Less than a third of the websites discussed psychological or emotional impacts patients might experience while considering or undergoing fertility preservation. Notably, few sites included patients’ gender identity and expression, which can impact fertility and parenthood, as well as long-term patient well-being. Websites that failed to address these issues by not mentioning transgender patients and/or not distinguishing between sex and gender could further isolate a cancer patient population that already experiences high rates of conditions like depression and other mental illnesses [54].

## Limitations, Strengths, And Future Directions:

As an initial step in a focused program of research, several limitations should be considered when interpreting the results. Our search terms and inclusion criteria focused on specifically identifying public education websites for AYA cancer patients considering fertility preservation; however, AYAs may choose to view clinical guidelines, online brochures, or other resources or websites that include information in narrative form. Similarly, focusing on general terms such as “teen” and “adolescent,” may have missed sites aimed at older AYA patients. However, these criteria add strength to the search for materials that AYA patients would likely encounter as our team approached our search using patient-friendly search terms and publically available records..

In terms of methods, the NCCN guidelines were also created to inform clinical encounters, not online media. Notably, we did not measure how many websites discussed testicular cryopreservation since the NCCN considers this treatment experimental at the time of our review[39]. The NCCN guidelines that informed our NCCN topic checklist were also created to inform clinical encounters, not necessarily delivered to patients through online media. But, we wanted to use the NCCN guidelines to evaluate the fertility preservation options covered on these websites using clinically relevant guidelines.

Notably, it was not within the scope of this review to assess whether patients or survivors were engaged in the development of currently-available websites. A key tenet of high-value health care is ensuring care is meaningful, accessible, and equitable by learning from patients about the full spectrum of outcomes that matter to each, and all, patients [55]. Further, desirable website features may have changed since Schiffman developed their measure in 2008; however, we felt it was important to evaluate the websites using AYA-generated criteria.

Strengths of our approach include that it balanced evidence-based systematic evaluation of multiple clinically- and AYA-relevant topics and features with AYA-derived criteria and a real-world focus on publicly-available websites used by patients and families. Results can inform and guide ongoing and future work designing high-quality online resources to address the gaps in AYA fertility preservation knowledge.

**Figure 3:**
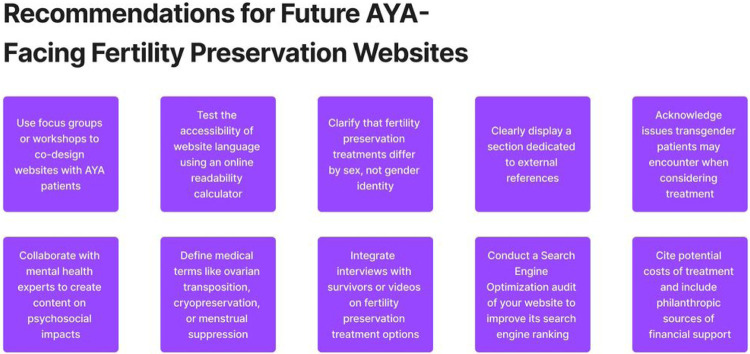
Recommendations

To address these gaps in health education for adolescent and young adult cancer patients, research is needed to develop and evaluate high-quality patient education websites tailored to young adults’ cognitive and communication needs. Higher quality content can be achieved through clear citations, unbiased writing, addressing a broad range of fertility preservation treatment options, and by including the financial, psychological, and emotional impacts of fertility care. Care should be taken to test and ensure appropriate reading levels, using clear definitions for complex medical terms. Additionally, incorporating desirable features such as videos or survivor narratives could increase engagement, and specifying terms related to sex and gender could improve inclusivity. Importantly, high-value health care approaches such as engaging diverse AYAs in multidisciplinary design teams should be used to ensure fertility preservation education materials focus on the health and psychological outcomes that matter to each and all adolescents and young adults. Finally, search engine optimization approaches can be used to ensure high-quality medical websites appear at the top of patients’ search results. For specific opportunities to consider incorporating these recommendations into AYA fertility preservation educational websites, see Fig. 3.

## Conclusions

While online patient education materials show much promise for improving younger patients’ awareness and education, there currently exists several significant gaps in the quality, comprehensiveness, readability, desirability, and inclusiveness of publicly-available fertility preservation websites for adolescents and young adults. Clinicians and researchers have a timely opportunity to engage AYAs to co-design high-quality, innovative patient education websites that foster improved patient knowledge and ability to engage in shared decision making about their oncofertility care.

## Figures and Tables

**Figure 2:**
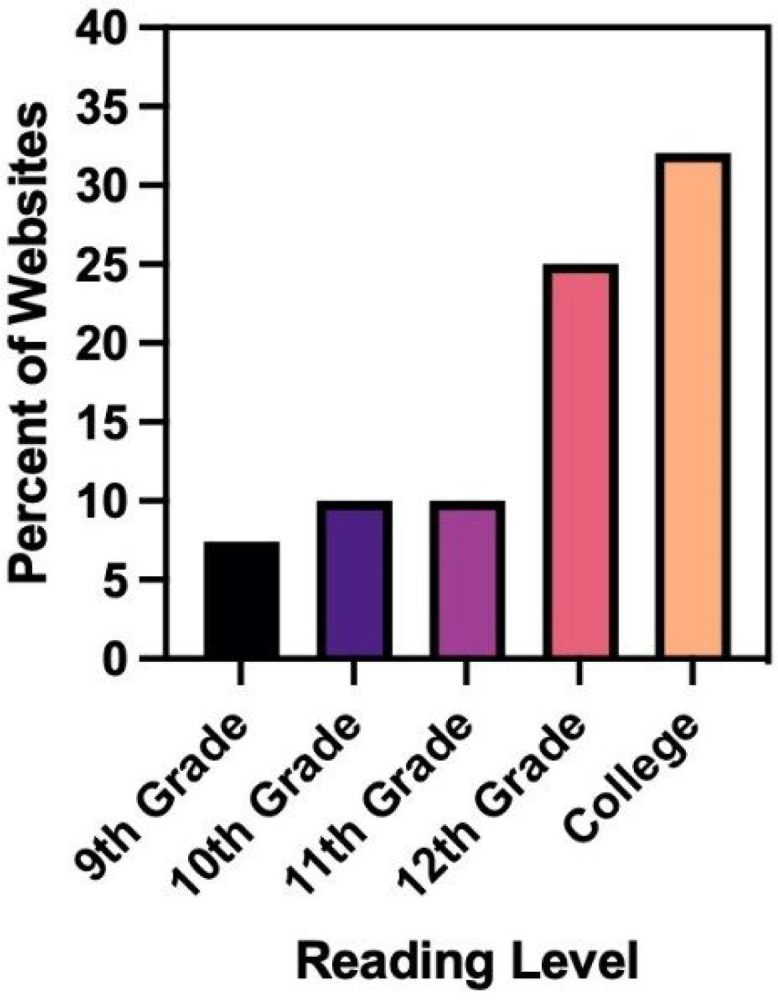
SMOG Reading Level Results

## Data Availability

The data generated by this project are available from corresponding author upon reasonable request.
